# Analysis of Normal Saliva

**Published:** 1878-06

**Authors:** B. F. Miller


### ANALYSIS OF NORMAL SALIVA.

BY B. F. MILLER, D. D. S.

There are several difficulties to contend with in the analysis of saliva, either normal or abnormal, which are as follows: First, it is usually used as fast as formed and is not very easily collected. Second, it decomposes the most rapidly of all fluids of the body with the exception of bile, that we must take into consideration the fact that it is decomposed. Third, the tests are so delicate for some of the constituents, that great care must be taken to have everything perfectly clean. By mixed saliva is meant that from the parotid, submaxillary, and sublingual glands, and the product from the mucous membrane of the mouth. It is of a pale bluish color, and when fresh has no odor; after standing for some time there is a deposit of mucous and pavement epithelium.

Methods of obtaining mixed saliva:

First. Thoroughly cleanse the mouth with cold water, open the mouth, press up under the chin and tickle the fauces with a feather so as to produce nausea but not vomiting.

Second. Cleanse the mouth as in the first and chew a glass stopper.

Third. Touch the tongue roof of the mouth and fauces with some irritating substance, such as citric acid.

Fourth. Tantalize the lower animals.

Fifth. The œsophagus of the lower animals may be cut as low down as possible and saliva obtained mixed with the food.

The second method is perhaps the best. Saliva from the separate glands is usually obtained from the lower animals by salivary fistula, tying ducts, etc. Saliva from the parotid gland may be obtained from the human subject by introducing a silver canula into the orifice of Steno's duct. The saliva from the parotid is perfectly clear and transparent; that from the sublingual will be the same as from the parotid, with the exception of KCy from parotid, and that from submaxillary is thick and viscid.

The specific gravity may vary from 1002 to 1009, but is usually between 1004 and 1006.

ANALYSIS OF NORMAL SALIVA.

Take some saliva and mix it with three times its bulk of distilled hot water and filter for the inorganic constituents.

*Chlorides* are always present in normal saliva in a combined state; if free HCl exists, the saliva is in an abnormal condition. Chlorides are often absent in pneumonia and other inflammatory affections; the reappearance of them is a sign of improvement.

Test.—Filtrate + $HNO_3$ + Ag $NO_3$ = a white precipitate of Ag Cl insoluble in acids but soluble in ammonia.

*Phosphates* may be excessive or deficient. They are in excess in exhaustive nervous diseases, in excessive brain work and in rickets, but deficient in pneumonia.

Test.—Filtrate + $HC_2H_3O_2$ + uranic acetate = a white precipitate of uranic phosphate.

Test for *Calcium.*— Filtrate + $(NH_4)_2C_2O_4 = CaC_2O_4$ which will seldom if ever be crystalline; if at all, it will be in the form of oxalates. It is insoluble in $HC_2H_3O_2$ but soluble in HCl.

Test for *Magnesium.*—Filtrate + $Na_2HPO_4 + NH_4Cl + NH_4OH$ = a white and flaky precipitate of $MgHPO_4$.

Potassium sulpho-cyanide is a constituent of parotid saliva, and can not always be found in mixed saliva, as it is not very abundant even in the parotid saliva. It is more abundant in the saliva obtained from children.

Test.—Filtrate + HCl + $Fe_2Cl_6$ = a blood-red solution of $Fe_2(CyS)_6$.

There is a difference of opinion as to whether carbonic acid is a normal or an abnormal constituent, but I believe it is abnormal. I think it is due to letting it stand in air, by so doing it receives $H_2CO_3$ from the air. Fresh saliva never contains it.

The action of normal saliva on Litmus paper is, I need hardly say, that of all new volatile alkali, viz: that it turns Litmus paper blue, and is not affected upon exposure to the air.

The principle organic constituents of normal saliva are albumen, mucin and ptyalin.

*Albumen.*—There are two tests for albumen. First, if saliva be strongly acidified with $HNO_3$ it becomes more turbid; if it then be boiled it becomes clear and of a yellow color, this showing the presence of albumen. Second, if to a portion of saliva a mixture of $HC_2H_3O_2$ and $K_4Cfy$ be added a white precipitate is produced.

*Mucin.*—Saliva owes its viscidity to mucin. Most of the viscid fluids of the body contain mucin.

Test.—To some saliva in a small beaker add $HC_2H_3O_2$ gradually stirring with a glass rod for fifteen minutes when the mucin will be coagulated in white stringy flakes.

*Ptyaline* is the fermentable substance of saliva. There are two methods of testing for it.

First. Collect a considerable amount of saliva and acidify it strongly with $H_3PO_4$, then add milk of lime until the mixture is faintly alkaline and filter. The ptyalin is now on the filter paper but contains many impurities. Remove the filter paper with contents to a clean beaker and add distilled water not to exceed the quantity of saliva originally used, and stir well, then filter again. The ptyalin is now in the filtrate and may be precipitated by the addition of absolute alcohol.

Second. As ptyalin exists already prepared in the salivary glands, it may be obtained from them more easily and in greater quantity than from the saliva. The parotid gland is the best to be used. Take the parotid gland of an ox or sheep and cut in very fine pieces, then place it in absolute alcohol and cork it up for twenty-four hours; then filter through cloth and squeeze the residue to get rid of as much alcoholic extract as possible. The cake thus obtained contains the ptyalin and should be placed in a beaker with glycerine, stirring frequently for twenty-four hours or more, then filter through cloth, then filter the filtrate through paper and add to the filtrate alcohol. The ptyalin will be precipitated in white flakes and may be collected and used as a ferment. I think the time will come when ptyalin will be obtained in this way and used in medicine to a considerable extent.

